# Recurrent Episodes of Paraphilic Behavior Possibly Associated With Olanzapine and Aripiprazole Treatment in a Patient With Schizophrenia

**DOI:** 10.3389/fpsyt.2020.00318

**Published:** 2020-04-20

**Authors:** Maria-Ioanna Stefanou, Debora Vittore, Ines Wolz, Stefan Klingberg, Dirk Wildgruber

**Affiliations:** ^1^ Department of Neurology & Stroke, and Hertie-Institute for Clinical Brain Research, Eberhard-Karls University of Tübingen, Tübingen, Germany; ^2^ Department of Psychiatry and Psychotherapy, Eberhard-Karls University of Tübingen, Tübingen, Germany; ^3^ Department of Clinical Psychology and Psychotherapy, Eberhard-Karls University of Tübingen, Tübingen, Germany

**Keywords:** schizophrenia, paraphilia, antipsychotic, olanzapine, aripiprazole

## Abstract

**Background:**

Hypersexual and paraphilic disorders have been frequently associated with concomitant psychiatric disorders, including schizophrenia. A growing number of published cases has recently indicated that hypersexual behavior may also arise in conjunction with treatment with second-generation antipsychotics. Although hypersexuality has been acknowledged as a possible side effect of antipsychotic treatment with partial dopamine agonists, including aripiprazole, only very few cases of olanzapine-associated hypersexuality have been reported in the literature.

**Case Presentation:**

A 29-year-old man presented with delusions of persecution and reference, auditory hallucinations, and negative symptoms, and was diagnosed with paranoid-hallucinatory schizophrenia. One and a half months after initiation of antipsychotic treatment with olanzapine, he developed compulsive sexual behavior and paraphilia, without signs of akathisia. After olanzapine discontinuation, a full remission of the hypersexual behavior was noted within one week, and treatment was switched to risperidone. Due to hyperprolactinemia, adjunct treatment with low-dose aripiprazole was initiated and a severe recurrence of identical hypersexual behavior occurred. The hypersexual behavior resolved completely within a week after aripiprazole discontinuation.

**Conclusion:**

This case illustrates that hypersexuality may be a rare adverse effect of treatment with second-generation antipsychotics. Although aripiprazole is a drug with a well-established risk for hypersexuality, the question of whether a causal association between hypersexuality and olanzapine exists remains currently unresolved. As the currently limited amount of available evidence precludes any definitive conclusions, additional research is warranted to delineate the possible neurobiological substrates of hypersexual and paraphilic disorders in patients treated with second-generation antipsychotics.

## Background

Hypersexual disorder is a relatively common clinical entity (1), characterized by excessive preoccupation with sexual fantasies, urges, and behaviors, and is associated with significant volitional impairment, disinhibition, impulsivity, compulsivity, or behavioral addiction (2). While hypersexual disorder refers to an excessive or disinhibited engagement in culturally adapted normophilic sexual behaviors, paraphilic disorders (e.g., exhibitionism, frotteurism, voyeurism, fetishism, sadism) (3) are characterized by abnormal or socially deviant forms of sexual preference and arousal, accompanied by clinically significant personal distress and psychosocial impairment (4, 5).

Both hypersexual and paraphilic disorders have been frequently reported in association with concomitant psychiatric diseases, including bipolar disorder (i.e., hypomanic or manic syndromes) and schizophrenia (6, 7). The dopamine hypothesis, which proposes that a preponderance of dopaminergic activity is implicated in psychosis propagation (8), is considered a plausible model for sexual disinhibition in psychotic patients (9). Interestingly though, a growing number of published cases has recently indicated that hypersexuality may also arise in conjunction with treatment with second-generation antipsychotics (SGA) (10–17).

Olanzapine is a widely prescribed SGA, which has been associated with sexual dysfunction (i.e., decreased libido, erectile dysfunction, impaired arousal, delayed orgasm) in up to 35% of treated patients (18–20). Nonetheless, a handful of recently published cases have reported olanzapine-associated hypersexuality (21–23), indicating that SGA-related sexual dysregulation remains currently ill-understood. Here, we present the case of an adult patient with schizophrenia, who presented with compulsive sexual behavior (CSB) and exhibitionism in association with olanzapine and aripiprazole treatment. A written informed consent was obtained from the patient.

## Case Presentation

A 29-year old male Caucasian patient presented with delusions of persecution and reference, auditory hallucinations, and negative symptoms, including avolition, flat affect, and social withdrawal (and symptom-duration of more than 3 months), and was diagnosed with paranoid-hallucinatory schizophrenia (ICD-10: F20.0). The patient had a previously unremarkable medical history and no history of substance abuse. The childhood developmental stages had been uneventful, but since adolescence he had been increasingly introverted and socially insecure. At the age of 29 years, he reported having had no previous relationships or sexual experiences. There were no psychiatric or neurologic diseases in the family history. On neuropsychological assessment, he presented mild cognitive deficits (i.e., impairment in concentration, attention, working memory, and executive function). A thorough diagnostic workup, including brain magnetic resonance imaging (MRI), cerebrospinal fluid analysis, electroencephalography, hematological investigations, and toxicological screening, was normal. The patient received antipsychotic treatment with risperidone (initially 5 mg/day orally, later switched to risperidone depot 37.5 mg/2 weeks intramuscularly). His symptoms improved rapidly, and at discharge, a complete remission of the psychosis had been achieved. Due to sleep disturbances, which the patient attributed to risperidone treatment, he decided to discontinue the medication directly after discharge. Seven months later, he was readmitted to the hospital with recurrent delusions of persecution, tactile, and visual hallucinations. Due to the severity of clinical presentation, he was administered haloperidol (20 mg/day orally), which led to complete remission of positive symptoms within 2 weeks. After thorough consideration of treatment options, a switch of treatment to olanzapine was decided (initially 15 mg/day orally, later switched to olanzapine depot 405 mg/4 weeks intramuscularly). In parallel to olanzapine titration, haloperidol was gradually tapered-off and eventually discontinued. One and a half months later, the patient was urgently admitted to the acute psychiatric ward with CSB having been charged with exhibitionism. Police records reported that the patient had sexually harassed several women in the preceding days (i.e., kissing them against their will or touching their genitalia). On admission day, the police reported that the patient had undressed himself and masturbated in public. On clinical examination, he showed uncontrolled sexual urges, overfamiliarity, and hypersexual behavior (e.g., with fixation on masturbation, sexual propositioning, harassment of non-consenting nursing and medical staff). Recurrent episodes of public exhibitionism were also recorded. Concurrently, he presented with disorganization, delusions of control (e.g., experiencing his body as being externally controlled) and delusions of telepathic communication skills, but no hallucinations were noted. There were no signs of akathisia. The patient had no prior arrests, indictments, or convictions, and his hypersexual behavior was in marked contrast to his premorbid personality of being shy and introverted. He reported having had increased libido and irresistible urge to masturbate during the preceding few weeks. There was no evidence of other substance abuse. The serum concentration of olanzapine was within the therapeutic range (29.1 μg/l). Because the CSB developed shortly after initiation of olanzapine, a causal relation to SGA treatment was suspected, and olanzapine was withdrawn. Treatment was changed back to haloperidol (20 mg/day orally) and initially supplemented with lorazepam (4 mg/day orally). Under this regimen, the hypersexual behavior diminished rapidly and disappeared after 1 week. The patient consented to rechallenge with risperidone (initially 5 mg/day orally, later switched to risperidone depot 37.5 mg/2 weeks intramuscularly), while haloperidol and lorazepam were tapered-off and eventually discontinued. Under treatment with risperidone, the patient developed a secondary hyperprolactinemia (prolactin 46.9 μg/l) and a decreased libido was suspected. To ensure treatment adherence, the combination of risperidone with low-dose aripiprazole (5 mg/day orally) was decided. Two weeks after initiation of aripiprazole, the patient presented with a severe CSB relapse with uncontrolled hypersexual behavior. Aripiprazole was immediately withdrawn and additive treatment with haloperidol (20 mg/day orally) and lorazepam (4 mg/day orally) was initiated. The hypersexual behavior diminished rapidly and disappeared after 1 week, while treatment was gradually changed to monotherapy with haloperidol (at discharge 75 mg/2 weeks intramuscularly with further scheduled tapering). The libido level of the patient remained normal, and no recurrence of psychotic symptoms was noted till his discharge from hospital, 5 months later.

## Discussion

The neurobiological mechanisms underlying regulation of sexual desire remain to date only partially understood. The interaction between brain monoaminergic (i.e., adrenergic and serotoninergic) receptors and sex hormones (i.e., testosterone) is considered pivotal for sexual responses and behaviors (2, 9). Enhanced dopaminergic neurotransmission is typically associated with sexual excitation, while enhanced serotoninergic neurotransmission with sexual inhibition (2, 24). Some of the brain regions implicated in physiological sexual arousal, attention, and motivation, include the hypothalamus, substantia nigra, ventral striatum, pallidum, amygdala, anterior insula, anterior cingulate cortex, inferior frontal cortex, fusiform gyrus, precentral gyrus, and parieto-occipital cortices (25–28).

In patients with primary hypersexual disorders, pathological alterations in frontal lobe, hypothalamus, amygdala, hippocampus, anterior cingulate cortex, and brain regions of the reward circuitry have been reported (25, 29). Secondary drug-induced hypersexuality, which is a well-established complication of dopamine-enhancing medications (e.g., antiparkinsonian drugs) (30, 31), has also been associated with enhanced activation in similar regions, including the ventral striatum, cingulate, and orbitofrontal cortices (32). As only few clinical cases of SGA-induced hypersexuality have been reported in the medical literature, no evidence on neurobiological or imaging correlates of SGA-induced hypersexuality exists (10–17). Nevertheless, an SGA-mediated increase in dopaminergic neurotransmission, a blockade of serotoninergic neurotransmission or a combined effect, have been suggested as possible pathways for SGA-induced hypersexuality (17).

Consistent with these hypotheses, several cases of hypersexual disorders under SGA with partial agonistic effects at dopamine D2 receptors [e.g., aripiprazole (10, 14, 15)] have been reported. Partial D2 agonists are considered to enhance dopaminergic drive at the mesolimbic system, thereby resulting in aberrant sexual excitation (10). Similar increases in dopamine activity in the mesocortical-dopamine pathways may be elicited by serotonin blockade (21). In particular, at receptor level, activation of the 5-HT2 receptor impairs sexual functioning, whereas 5-HT1A receptor stimulation facilitates sexual function (9). Thus, drugs with properties of 5-HT1A partial agonism and 5-HT2 partial antagonism, such as aripiprazole, may induce hypersexuality (10). In addition to the receptor profiles, the reduced risk for hyperprolactinemia of these drugs may facilitate emergence of hypersexual behaviors (33).

Conversely, the receptor binding profile of olanzapine is consistent with antidopaminergic activity (high affinity for dopamine D1, D2, D4 receptor subtypes), antiserotonergic activity (high affinity for serotonin (5-HT2A, 5-HT2C, 5-HT3 receptor subtypes), anti-α1-adrenergic, and antimuscarinic activity (34). The blockade of serotoninergic neurotransmission, that results in increase in dopamine activity in the mesocortical dopamine pathways, is considered a possible underlying mechanism for occurrence of hypersexuality in patients under olanzapine monotherapy (21). Longitudinal treatment with olanzapine has also been associated with enhancement in dopaminergic activity of the prefrontal cortex and attenuation of amygdala reactivity in emotional processing (35). However, the association of these effects and sexual function in patients with schizophrenia has not been investigated.

Another etiological hypothesis is that olanzapine-mediated α1-adrenergic and anticholinergic antagonism in the peripheral nervous system may also affect erectile, orgasmic, and ejaculatory function, with two cases reporting olanzapine-induced priapism (36) and spontaneous ejaculations (37) in adult and pediatric patients, respectively. Furthermore, one reported case of a patient presenting with exhibitionism (i.e., masturbation in public) after intramuscular application of olanzapine, described concomitant akathisia (i.e., motor restlessness), suggesting a possible overlap between hypersexuality and akathisia (22).

On the other hand, only very few cases of risperidone-induced hypersexuality have been reported in the medical literature (16), while results from a randomized clinical trial showed less sexual dysfunction in patients treated with olanzapine compared to risperidone (38). Moreover, accumulating epidemiological evidence suggests that among the currently available antipsychotics, risperidone is the antipsychotic most frequently related to hyposexuality, amenorrhoea, and galactorrhoea, and is closely followed by haloperidol (39, 40).

Here we presented a patient with schizophrenia, who developed CSB and paraphilic behavior (without signs of akathisia) shortly after initiation of treatment with olanzapine. Although a causal relation between the behavioral changes and olanzapine cannot be definitely established, the temporal association between the emergence of hypersexuality and the initiation of SGA treatment with olanzapine, as well as the full remission of the hypersexual behavior 1 week after olanzapine discontinuation suggest a possible link between olanzapine and hypersexuality. Additionally, the recurrence of identical CSB symptoms shortly after initiation of aripiprazole (i.e., a drug with well-established risk for hypersexuality) and the resolution of the hypersexual behavior 1 week after aripiprazole discontinuation support the hypothesis of a drug-related hypersexuality in this patient. Nonetheless, as during the phase of CSB concomitant psychotic symptoms (e.g., delusions and disorganization) were noted, it could be possible that SGA simply unmasked or failed to control (e.g., after discontinuation of haloperidol and lorazepam) hypersexual symptoms in the context of psychosis.

Crucially, “overshooting” phenomena, including new, severe positive psychotic symptoms (41), along with paradoxical effects, including symptom worsening after switch of treatment or treatment discontinuation/reduction (42, 43) have been previously reported, and are currently considered to reflect a drug-induced dopamine hypersensitivity in schizophrenic patients. As the manifestation of CSB in our patient occurred on both occasions (i.e., under olanzapine and under aripiprazole) after modification of the antipsychotic treatment, an antipsychotic-induced hypersensitivity psychosis or a paradoxical effect (e.g., serotonin withdrawal syndrome with autonomic and sexual symptoms) should be considered in the differential diagnosis (41, 42).

To the best of our knowledge, only four previously published reports have discussed the occurrence of hypersexuality under olanzapine treatment (21–23, 36). Thus, if an association between olanzapine and hypersexuality exists, the probability of this side-effect should be considered very rare. Pointing towards a causal relation between hypersexuality and olanzapine, a previous report of a child, who developed CSB and excessive masturbation after initiation of olanzapine treatment, describes a complete resolution of the hypersexual behavior 1 week after olanzapine discontinuation and reemergence of CSB after rechallenge with olanzapine (21). For this reason, in our patient, a rechallenge was not attempted and switch of SGA treatment was decided. Interestingly, besides emergence of hypersexuality, a *de novo* manifestation or exacerbation of CSB in patients with schizophrenia treated with olanzapine has also been previously reported (44, 45). In accord with all reported cases of olanzapine-induced hypersexuality, the hypersexual behavior of our patient resolved completely within few days after olanzapine discontinuation (21–23, 36). Similar was the course of the CSB after discontinuation of aripiprazole.

In conclusion, this case indicates that hypersexuality may occur under SGA treatment with olanzapine and aripiprazole. Although aripiprazole is a drug with a well-established risk for hypersexuality, the question of whether a causal association between hypersexuality and olanzapine exists remains currently unresolved. In our patient, an alternative explanation could be that olanzapine unmasked or failed to control hypersexual symptoms in the context of psychosis. Nonetheless, based on this case, we suggest, that it would be advisable to consider modification of SGA treatment if hypersexuality under olanzapine arises. Furthermore, in patients presenting with hypersexual behavior under any SGA (including olanzapine), avoidance of subsequent treatment with partial dopamine agonists, including aripiprazole, might be advisable. As the currently limited amount of available evidence precludes any definitive conclusions, additional research is warranted to delineate the true incidence and the possible neurobiological substrates of hypersexual and paraphilic disorders in patients treated with SGA medication.

## Ethics Statement

Written informed consent was obtained for the publication of this case report.

## Author Contributions

M-IS and DW created concept and design of the study, and jointly wrote the manuscript. DV, IW, and SK provided the clinical data and critically reviewed the manuscript.

## Conflict of Interest

The authors declare that the research was conducted in the absence of any commercial or financial relationships that could be construed as a potential conflict of interest.
